# Fam65b Phosphorylation Relieves Tonic RhoA Inhibition During T Cell Migration

**DOI:** 10.3389/fimmu.2018.02001

**Published:** 2018-09-11

**Authors:** Laura Megrelis, Elyas El Ghoul, Federica Moalli, Margaux Versapuech, Shamir Cassim, Nora Ruef, Jens V. Stein, Marianne Mangeney, Jérôme Delon

**Affiliations:** ^1^Infection, Immunity, Inflammation, Inserm, U1016, Institut Cochin, Paris, France; ^2^CNRS, UMR8104, Paris, France; ^3^Université Paris Descartes, Sorbonne Paris Cité, Paris, France; ^4^Theodor Kocher Institute, University of Bern, Bern, Switzerland

**Keywords:** Rho GTPases, T cell migration, chemokine signaling, mouse model, phosphoproteins

## Abstract

We previously identified Fam65b as an atypical inhibitor of the small G protein RhoA. Using a conditional model of a Fam65b-deficient mouse, we first show that Fam65b restricts spontaneous RhoA activation in resting T lymphocytes and regulates intranodal T cell migration *in vivo*. We next aimed at understanding, at the molecular level, how the brake that Fam65b exerts on RhoA can be relieved upon signaling to allow RhoA activation. Here, we show that chemokine stimulation phosphorylates Fam65b in T lymphocytes. This post-translational modification decreases the affinity of Fam65b for RhoA and favors Fam65b shuttling from the plasma membrane to the cytosol. Functionally, we show that the degree of Fam65b phosphorylation controls some cytoskeletal alterations downstream active RhoA such as actin polymerization, as well as T cell migration *in vitro*. Altogether, our results show that Fam65b expression and phosphorylation can finely tune the amount of active RhoA in order to favor optimal T lymphocyte motility.

## Introduction

Rho GTPases are involved in signaling pathways that regulate very broad biological functions such as cell development, differentiation, and cytoskeletal alterations, in both physiological and pathological conditions in many different cell types (1–3), including in immune cells (4–7).

Rho GTPases are considered as molecular switches because they oscillate between an inactive GDP-bound state and an active GTP-bound form. This rapid cycle of activation-inactivation is made possible by two classical families of regulatory proteins: Guanine nucleotide exchange factors (GEFs) stimulate the GDP/GTP exchange, whereas GTPase activating proteins (GAPs) favor GTP hydrolysis (8). In addition, another family of proteins called GDP dissociation inhibitors (GDI) regulates the cytosol / plasma membrane localization of Rho GTPases (8).

In addition to these classic Rho GTPases regulators, a limited number of RhoA inhibitors that do not contain typical GAP domains, have been described more recently. These include a vaccinia virus -encoded protein called F11L (9) and a mammalian protein called Memo (10).

We were the first to show that the poorly studied Fam65b protein was also an atypical RhoA partner that binds RhoA directly and inhibits its activation in T lymphocytes (11). This role of Fam65b was further confirmed in neutrophils (12). Interestingly, Fam65a, an orthologue of Fam65b, was also shown to decrease RhoA-GTP levels in HeLa cells (13). Although no study has reported any role for Fam65c so far, the current experimental evidence gathered for the Fam65a and Fam65b members, point toward a role for proteins of the Fam65 family in fixing a low RhoA activity level in resting cells.

However, although resting T cells exhibit low RhoA-GTP levels in part through Fam65a- and/or Fam65b-mediated inhibition of RhoA activation, we know that many stimuli received by cells are able to activate RhoA in a time frame when Fam65b is still highly expressed. Thus, one might wonder which mechanisms are at play in cells to allow the rapid neutralization of the brake imposed by Fam65b on RhoA activation.

Here, we show that chemokine stimulation induces a rapid Fam65b phosphorylation which decreases Fam65b-RhoA binding, allows cytosolic localization of Fam65b, and favors RhoA activation. This process is functionally important to adjust RhoA activation levels because we show that overexpressing Fam65b or knocking-out its expression, impair T lymphocyte migration both *in vitro* and *in vivo*.

## Results

### Fam65b inhibits intranodal T lymphocyte migration

In order to determine whether Fam65b controls T cell migration *in vivo*, we generated a conditional murine model in which Fam65b was specifically deleted in the T lymphocyte lineage (Supplementary Figure 1). Fam65b was efficiently deleted in knock-out (KO) mice as shown by the absence of both Fam65b isoforms 1 and 2 in thymocytes and mature T cells (Supplementary Figure 2A). As expected, the deletion of Fam65b was only observed in the T cell lineage because non-T cells obtained from KO mice expressed normal levels of Fam65b (Supplementary Figure 2B). KO mice were perfectly viable, fertile and did not exhibit any alteration in thymocyte and T lymphocyte numbers in all the primary and secondary lymphoid organs studied (Supplementary Figure 2C).

**Figure 1:**
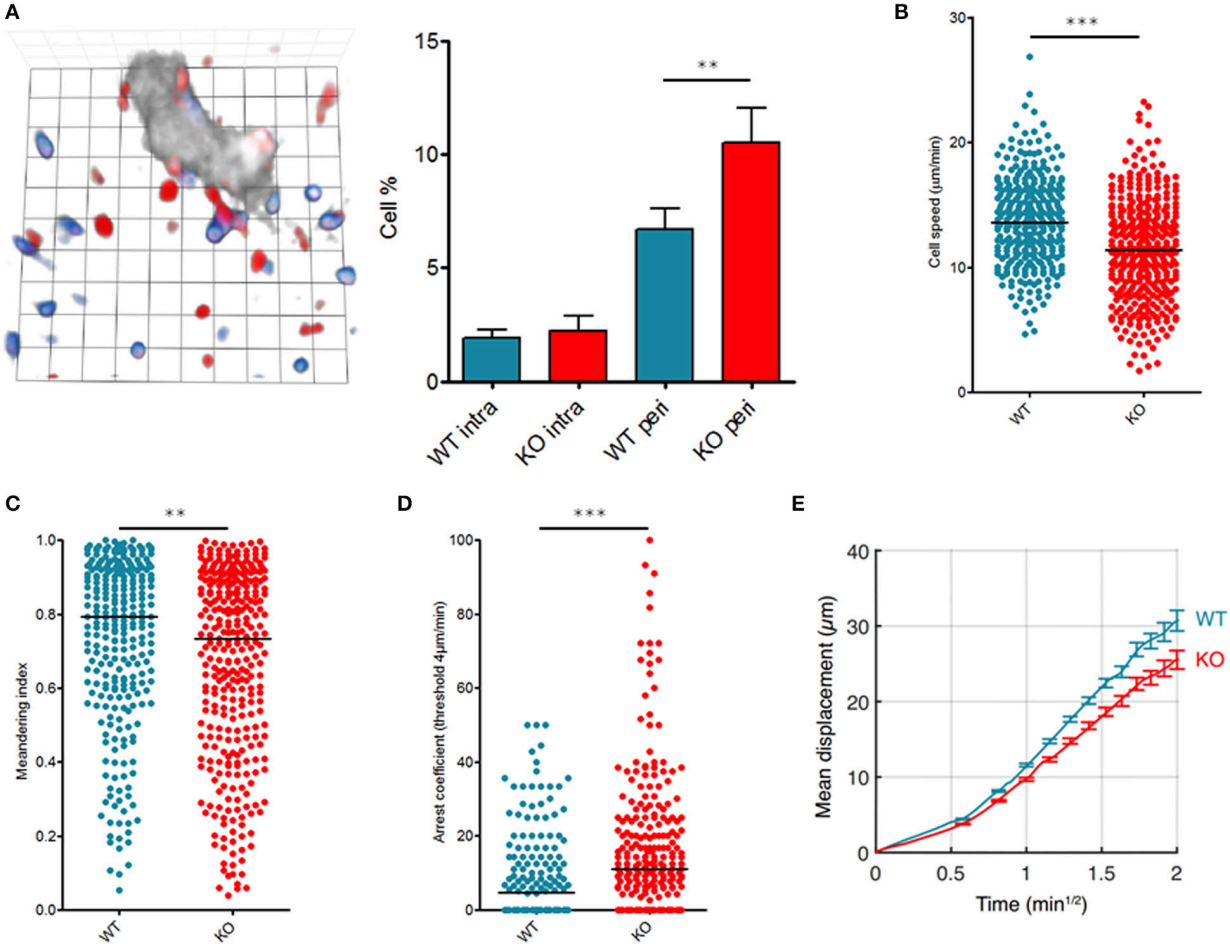
Fam65b inhibits T cell migration *in vivo*. T lymphocytes purified from WT or Fam65b KO mouse peripheral lymph nodes were loaded with distinct fluorescent probes and injected at a ratio of 1:1 into a recipient wild-type mouse. **(A)** Left: Microscopic field showing WT (blue) or KO (red) T cells together with HEV staining (gray). 1 unit scale = 8.6 μm. Right: Percentage of lymph node WT or KO T cells retained near HEV in the perivascular region (peri) or located inside HEV (intra) as described in (15). Distribution of single T cell speeds **(B)**, meandering indexes **(C)** and arrest coefficients calculated for a threshold of 4 μm/min **(D)** are shown. **(E)** Average displacement of cells according to the square root of time. The leading coefficient of this line is the coefficient of motility. **p < 0.01, ***p < 0.001.

First, we identified the micro-anatomical location of transferred T cells by performing ultra-short homing assays followed by 3D lymph nodes reconstructions (14). Although similar numbers of WT and KO T cells were observed inside high endothelial venules (HEV), KO T cells exhibited a bias compared to WT cells: KO T cells were more prone to reside close to HEV than their WT counterparts (Figure 1A).

We next analyzed intranodal migration of wild-type (WT) or Fam65b^KO^ T cells using two-photon microscopy of anesthetized mice as reported (16, 17). 24 h after injection of a mix of fluorescently labeled WT and KO T cells, both populations were compared for their single cell speed and the straightness of their migratory trajectories into the lymph nodes parenchyma in homeostatic conditions. Both the speed (Figure 1B) and meandering index (Figure 1C) of KO T cells were reduced indicating that in the absence of Fam65b, T lymphocytes migrate more slowly and use less straight paths. Fam65b KO T cells also exhibited a higher tendency to arrest (Figure 1D). Accordingly, because of this reduced migration speeds and more frequent changes in directionality, Fam65b KO T cells showed a significantly lower motility coefficient (Figure 1E).

### Fam65b restricts spontaneous RhoA activation *in vivo*

Based on our previous results and other studies showing that Fam65a and Fam65b inhibit RhoA activity *in vitro* (11–13), we next determined whether resting Fam65b KO T cells exhibit alterations in RhoA-GTP levels.

**Figure 2:**
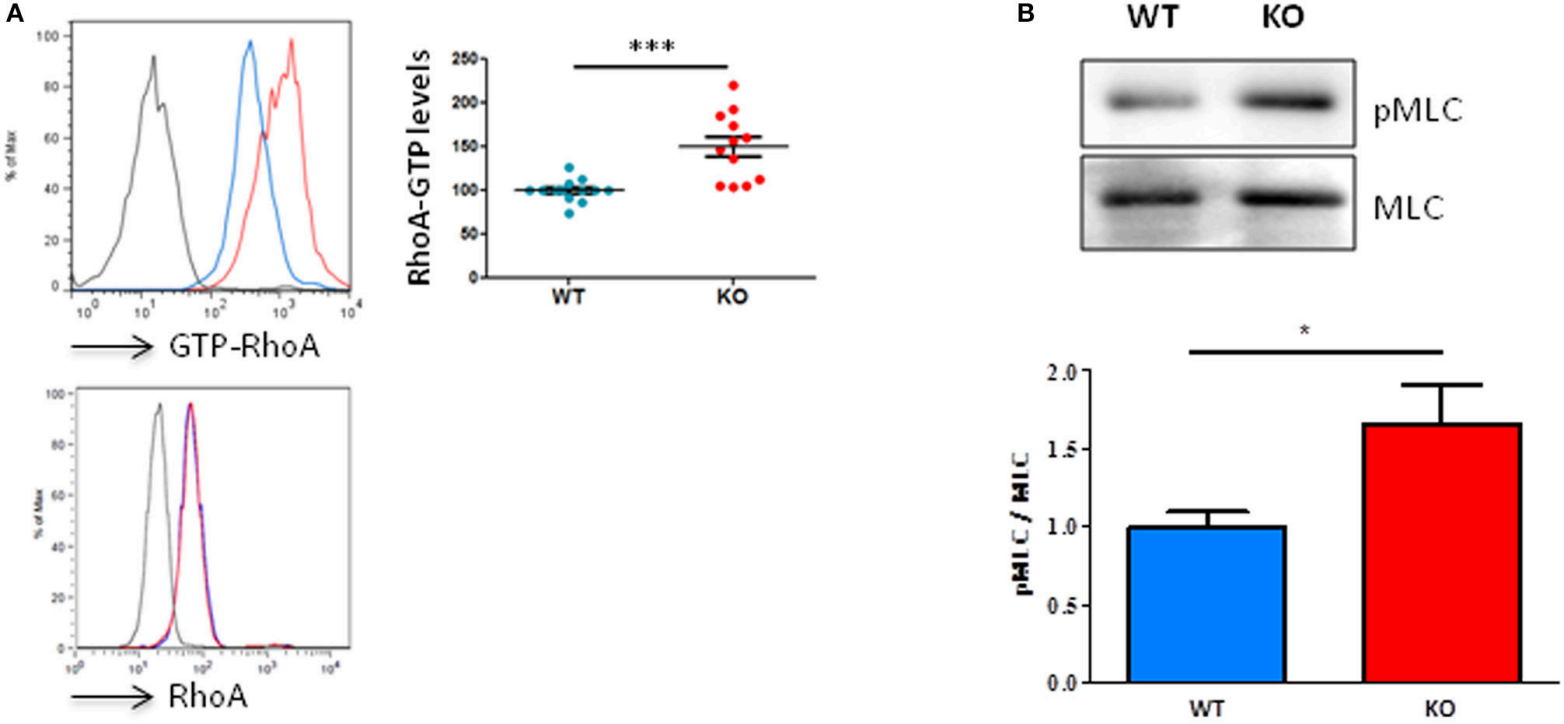
Fam65b KO T cells exhibit an exacerbated RhoA signaling pathway. **(A)** Top left panel: Example of detection of the amount of RhoA-GTP by flow cytometry in lymph node T lymphocytes from WT (blue) or Fam65b KO (red) mice. Top right panel: RhoA-GTP levels from eight independent experiments are shown. The intensity of the RhoA-GTP staining obtained in each experiment is normalized to the average values of WT mice. Bottom panel: The detection of the total amount of RhoA in T cells shown by flow cytometry shows no difference between WT and Fam65b KO mice. **(B)** Top: After purification of T lymphocytes from WT or Fam65b KO mice, expression of phospho-MLC (pMLC) and total MLC was analyzed by Western blot. Bottom: Quantification of the pMLC/MLC ratio measured in three independent experiments. *p < 0.05, ***p < 0.001.

By using an antibody that specifically recognizes active RhoA, we were able to show, in homeostatic conditions, that unchallenged resting T lymphocytes from Fam65b^KO^ mice exhibit a significant higher basal level of RhoA-GTP compared to T cells purified from control WT littermates (Figure 2A, top). This difference was not due to changes in total RhoA levels (Figure 2A, bottom). Therefore, these results show that Fam65b exerts a tonic inhibition on RhoA activity in primary resting mouse T lymphocytes.

We next aimed at determining whether such a high level of active RhoA observed in Fam65b^KO^ T cells could lead to downstream modifications of the RhoA signaling pathway. We focused our analysis on the phosphorylation levels of myosin light chains (MLC), as this process is known to be triggered by the Rho-associated kinase ROCK, a key regulator of actin organization and thus a regulator of cell migration downstream active RhoA (18). In agreement with the higher RhoA-GTP levels found in Fam65b^KO^ T cells, our results clearly showed that KO T cells have a higher content in active P-MLC than their WT counterparts (Figure 2B).

Altogether, the aberrant and constitutively high levels of active RhoA measured in resting KO T cells are most likely responsible for the defects in intranodal migration reported here. These results indicate that too much of RhoA-GTP inhibits T cell migration, and show that Fam65b restricts spontaneous RhoA activation in resting T cells. Nevertheless, in normal conditions when WT T cells express Fam65b, it is well known that RhoA becomes activated upon chemokine stimulation (11, 19). It is not clear how the brake that Fam65b exerts on RhoA activation can then be relieved to achieve an increase in RhoA-GTP levels upon chemokine receptor signaling. We previously reported that the strong and constitutive RhoA-Fam65b interaction in resting T cells is transiently disrupted upon CCL19 stimulation (11). Thus, we have next attempted to delineate further the molecular mechanisms responsible for this RhoA-Fam65b dissociation.

### Fam65b phosphorylation upon chemokine stimulation inhibits RhoA-Fam65b interaction

**Figure 3:**
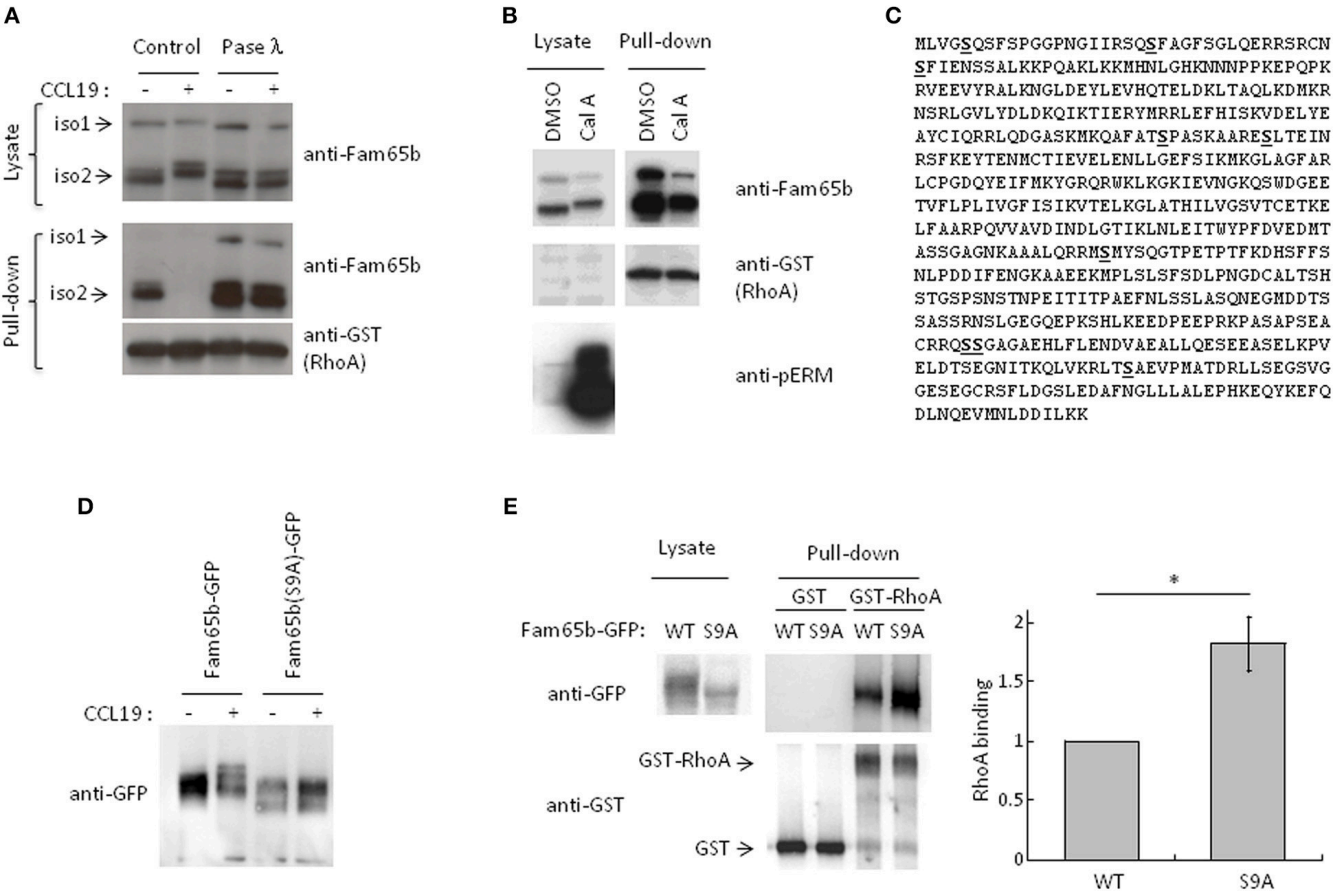
CCL19 stimulation phosphorylates Fam65b and controls Fam65b-RhoA interaction. **(A)** T cells were stimulated or not with 200 ng/ml of CCL19 for 2 min. The resulting lysates were incubated without (Control) or with Phosphatase (Pase) λ for 45 min. A fraction of each samples was then analyzed by Western blot immunoblotting, using an anti-Fam65b antibody that detects both Fam65b isoforms 1 and 2. The other lysate fractions were used for GST-Pulldown experiments with recombinant GST-RhoA. Fam65b immunoblotting of pull-down samples allows detection of the amount of Fam65b bound to RhoA. **(B)** PBT were treated with 200 nM Calyculin A (Cal A) or vehicle (DMSO) for 10 min. A fraction of the whole cell lysates was directly analyzed by Western blot, whereas another fraction was used for a GST Pull-down experiment with recombinant RhoA, as previously described. Antibodies against Fam65b, GST and pERM were used in these immunoblots. **(C)** Amino acid sequence of Fam65b isoform 2. The serine residues identified by phosphoproteomics are underlined in bold. **(D)** Western blot analysis of WT and S9A Fam65b-GFP proteins in PBT unstimulated or stimulated with 200 ng/ml CCL19 for 2 min. **(E)** Left: Lysates from 239T cells transfected with WT or S9A Fam65b-GFP constructs were incubated with beads containing recombinant GST-RhoA for GST Pull-down experiments. The amount of Fam65b bound to the beads was detected by anti-GFP immunoblot. Right: Quantification of the binding of WT or S9A Fam65b-GFP. Fam65b band intensities in the pull-down fraction were normalized to their expression in the total lysate. For each of the four independent experiments performed, the amount of binding of Fam65b-GFP WT to RhoA was set to 1. *p = 0.04.

Western blot analysis of protein samples obtained from lysates of T cells stimulated with CCL19 or CXCL12 showed that both Fam65b isoforms migrated with a higher apparent molecular weight in stimulated conditions (Figure 3A, Supplementary Figure 3). These shifts were completely abrogated when the protein lysates were treated with phosphatase λ to remove all phosphate groups from the serine, threonine and tyrosine amino acids. We even observed that Fam65b migrated slightly lower than in the non-treated cells after the phosphatase λ treatment, including in the unstimulated cells (Figure 3A, top). These results show that Fam65b phosphorylation is strongly increased upon chemokine stimulation in T lymphocytes. They also suggest that Fam65 is already phosphorylated at a low level in unstimulated resting T cells.

Using pull-down assays run in parallel, we next compared the amount of RhoA associated with Fam65b in these different conditions. We observed that upon CCL19 stimulation, RhoA binding to Fam65b was lost. In contrast, phosphatase λ treatment strongly increased this binding, suggesting that non-phosphorylated Fam65b proteins are the most efficient RhoA binders. Weakly phosphorylated Fam65b present in resting T cells could also bind RhoA, although to a lesser extent.

Altogether, these results suggest a role for Fam65b phosphorylation in controlling the ability of Fam65b to bind RhoA.

This result was reinforced by additional data using a specific Ser/Thr phosphatase inhibitor, Calyculin A (Figure 3B). Ser/Thr phosphorylated proteins such as the ERM (Ezrin/Radixin/Moesin) proteins were used here as a positive control. As previously described (20), pERM levels were dramatically increased in T lymphocytes treated with this drug. We observed that both Fam65b isoforms exhibit a marked shift toward higher apparent molecular weight species with Calyculin A, suggesting that in resting T cells, Fam65b can be constitutively phosphorylated on Ser and Thr residues. Importantly, pull-down assays performed in parallel to compare the amount of RhoA binding to Fam65b in cells treated or not with Calyculin A, showed a clear decrease of RhoA binding with the drug (i.e., in conditions where Fam65b is highly phosphorylated). This result therefore confirms a negative correlation between the degree of Fam65b phosphorylation and its ability to bind RhoA.

We next aimed at identifying the Fam65b amino acids that can be phosphorylated in physiological conditions using an unbiased phospho-proteomic analysis. For this assay, we focused our interest on the most abundant and shortest isoform 2 of Fam65b whose sequence is fully included in the longer isoform 1. We have also previously shown that this isoform exhibits a stronger affinity to RhoA than isoform 1 (11). We were able to map nine serine residues whose probability of phosphorylation was close to 100%, at positions 5, 21, 37, 166, 175, 341, 473, 474, and 523 (Figure 3C). No threonine or tyrosine residues could be identified in this assay.

Next, in order to determine the role of these residues in Fam65b phosphorylation and its ability to bind RhoA, we generated a mutant form of Fam65b called Fam65b(S9A) in which all nine identified serine residues were mutated into alanine. This construct was fused to GFP. We first checked whether the shift of the molecule in resting T cells transfected with the Fam65b(S9A)-GFP construct and stimulated with CCL19 was still apparent. As shown in Figure 3D, no shift could be observed upon stimulation, contrary to the WT Fam65b-GFP protein.

We next used this mutant to determine to what extent Fam65b phosphorylation impacts on its ability to bind RhoA. For this purpose, plasmid constructs encoding for Fam65b WT or S9A were transfected into the 293T cell line that does not express endogenous Fam65b. The ability of Fam65b WT and S9A to bind RhoA was tested in pull-down assays (Figure 3E). The results show that the Fam65b(S9A) mutant binds RhoA more strongly than the wild-type form of the molecule. Quantification of the amount of Fam65b bound to RhoA in four independent experiments indicated a 1.8-fold increase with the mutant compared to the WT Fam65b protein. These results indicate that Fam65b phosphorylation on serine residues upon CCL19 T cell stimulation is responsible for the dissociation of the Fam65b-RhoA complex.

### Control of Fam65b localization by phosphorylation and RhoA binding

It is well documented that RhoA shuttles between the cytosol, where its association with GDI proteins maintains it in an inactive state, and the plasma membrane, where it gets activated by GEFs (1). Because Fam65b binds RhoA and because the above results show that CCL19 stimulation modifies the phosphorylation status of Fam65b, we next wondered whether this post-translational modification also alters Fam65b subcellular localisation. Using a cell fractionation assay, we therefore studied whether CCL19 stimulation of T lymphocytes that affects Fam65b phosphorylation levels, also changes its distribution.

**Figure 4:**
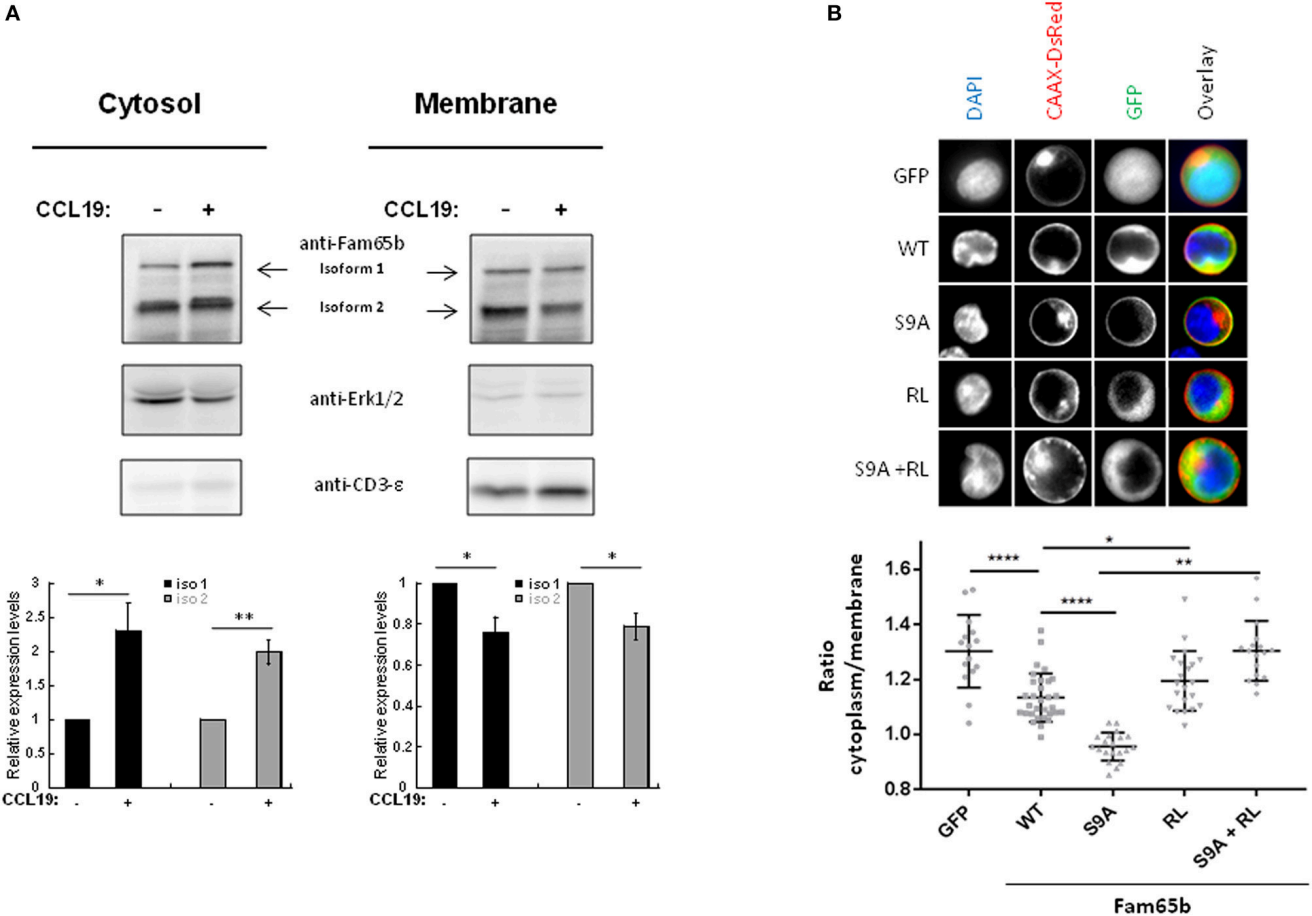
Phosphorylation controls Fam65b shuttling between the plasma membrane and the cytosol. **(A)** Top: PBTs were stimulated or not with 200 ng/ml CCL19 for 2 min before performing a cell fractionation assay. The obtained cytosolic and membranous fractions were then analyzed by Western blot using an anti-Fam65b antibody as well as the following antibodies directed against: Erk1/2 and CD3-ε used as specific markers for the cytosol and the membranes, respectively. Bottom: Quantification of the levels of expression of Fam65b isoforms 1 and 2 after normalization to controls of each compartment for each time condition (means ± SE from five independent experiments). **(B)** Top: CEM cells were transfected with the GFP expression vector alone, Fam65b-GFP WT, Fam65b(S9A)-GFP, the non-RhoA binding mutant Fam65b(RL)-GFP or the double mutant Fam65b(S9A, RL)-GFP together with pCAAX-DsRed to stain the plasma membrane in red. These cells were then labeled with DAPI (blue) to stain the nucleus. The fluorescence microscopy images shown were randomly acquired at 60X magnification. Bottom: Quantification of GFP intensity in the cytoplasm/plasma membrane of CEM cells. Each symbol corresponds to the analysis of one cell. A total of 15–35 cells per transfection condition were included in this quantification. *p < 0.05, **p < 0.01, ****p < 0.0001.

Primary T lymphocytes were stimulated or not with CCL19 and cytosol or membrane fractions were isolated (Figure 4A). The degree of purity of each fraction was tested by blotting for proteins expressed specifically in each compartment: Erk1/2 and CD3-ε, respectively. Upon CCL19 stimulation, quantifications of the amount of Fam65b present in the cytosol exhibited an increase, with a concomitant decrease in the membranous fraction (Figure 4A, bottom). The same results were obtained for both Fam65b isoforms 1 and 2.

This result raises the possibility that the Fam65b phosphorylation observed upon CCL19 stimulation could be responsible *per se* for the cytosolic accumulation of Fam65b. To address this question directly, we analyzed the subcellular localization of the non-phosphorylated Fam65b(S9A)-GFP mutant transfected in the CEM T cell line that does not express endogenous Fam65b. CEM cells exhibit a larger cytosol than primary T cells and are thus more suitable for visualizing the different subcellular regions by imaging techniques. Cells were co-transfected with a CAAX-DsRed construct and stained with DAPI to visualize in parallel the plasma membrane and the nucleus, respectively. Images of individual cells are shown (Figure 4B, top). The most striking feature that could be obviously evidenced in these cell pictures was the strong membrane localization of the non-phosphorylated S9A mutant of Fam65b. By quantifying the intensity of the GFP signal present in the cytosol and at the membrane in individual cells, we were able to calculate a cytoplasm/membrane ratio for each individual cell. Results showed that compared to GFP, this ratio was lower for the WT Fam65b-GFP form indicating that a fraction of Fam65b localizes at the plasma membrane, in accordance with the cell fractionation assay shown previously (Figure 4A). Most strikingly, this cytoplasm/membrane ratio was strongly decreased with the S9A mutant. This result indicates that Fam65b phosphorylation favors its cytosolic distribution. In addition, we and others previously showed that Fam65b mutations of Arg151 and Leu152 in Ala inhibited RhoA binding (12, 21). When we introduced such RL mutation in both WT and S9A Fam65b forms, one could observe that the corresponding mutants adopt a cytosolic distribution. Altogether, this indicates that the preferential association of Fam65b to the plasma membrane requires interaction with RhoA.

### Functional analysis of Fam65b actions downstream RhoA

In order to test the functional consequences of Fam65b and its mutated forms on RhoA activity, we used the HBMEC cell line transfected with the different Fam65b constructs. Indeed, in these cells that do not express endogenous Fam65b, F-actin stress fibers have been reported to be directly under the control of RhoA (22). Thus, we analyzed the capacity of various Fam65b mutants to interfere with the formation of F-actin stress fibers. Cells were stained with phalloidin to visualize the actin cytoskeleton network (Supplementary Figure 4, top) and the number of stress fibers per cell was quantified (Supplementary Figure 4, bottom left). Control cells transfected with GFP alone exhibited an average of 13 stress fibers per cell. By contrast, cells expressing the wild-type Fam65b form exhibited a marked decrease of actin stress fibers, with the Fam65b(S9A) mutant inhibiting almost completely their formation. Interestingly, the addition of the RL mutation in both WT and S9A Fam65b constructs blocked the inhibition of Fam65b on actin stress fibers generation. Therefore, this indicates that Fam65b WT and S9A need to interact with RhoA in order to inhibit the formation of stress fibers. In addition, the quantification of the total amount of F-actin in these different conditions clearly indicates that cells transfected with the RhoA binding competent forms of Fam65b exhibit a strong reduction in actin filaments staining (Supplementary Figure 4, bottom right). This indicates that the loss of actin stress fibers is not compensated by the generation of another type of actin structure.

Generally, we show here that, by inhibiting RhoA activity, Fam65b affects two major downstream RhoA pathways responsible for cytoskeletal alterations: actin polymerization and stress fibers formation in adherent cells.

We next investigated the possible functional consequences of these cytoskeletal alterations induced by Fam65b on T cell migration. Once again, we used the CEM cell line to test the effect of expression of the different mutants of Fam65b. We first checked whether optimal CEM migration requires a fully functional RhoA signaling pathway. For this, we used the well-documented ROCK inhibitor Y27632 (23), as ROCK is a key downstream target of RhoA to induce cell migration in various cellular models (7). As shown in Supplementary Figure 5, dampening ROCK activity in CEM cells strongly suppressed their migration induced by the chemokine CXCL12.

**Figure 5:**
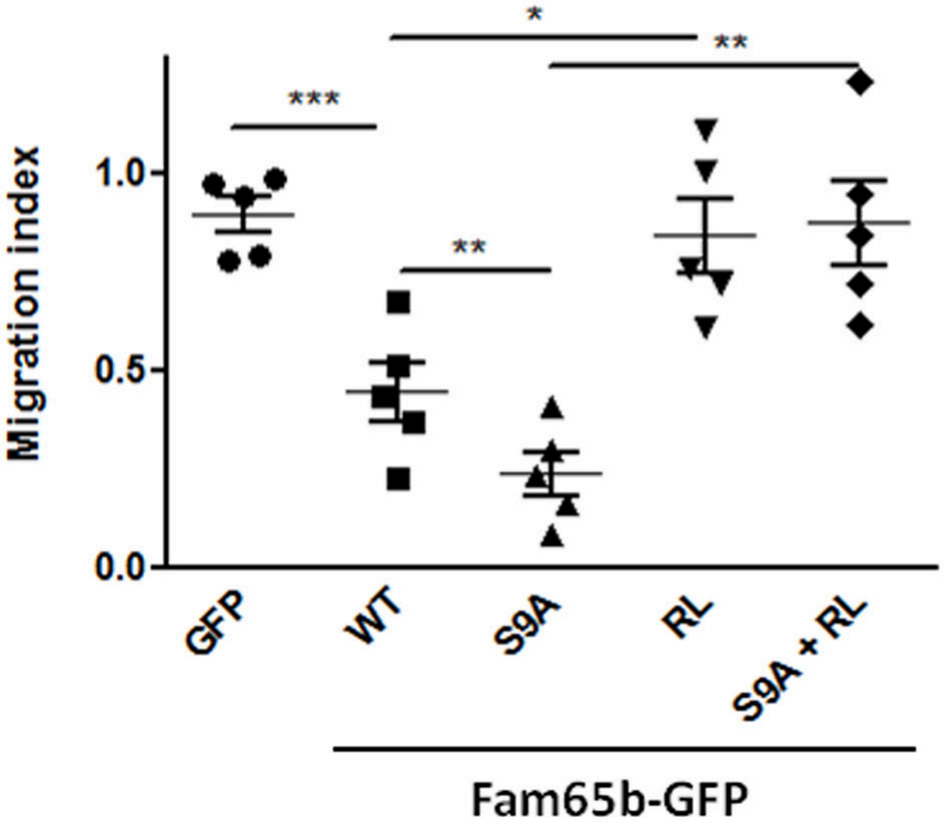
Fam65b inhibits the RhoA signaling pathway and T cell migration *in vitro*. Quantification of the Transwell migration index in five independent Transwell assays performed on CEM cells transfected with the indicated GFP-tagged constructs. Each symbol corresponds to an independent experiment. *p < 0.05, **p < 0.01, ***p < 0.001.

Using this read-out, we thus checked whether expression of WT Fam65b or Fam65b(S9A) had an effect. Both inhibited T cell migration very efficiently (Figure 5), especially the S9A mutant (70 vs. 45% inhibition, respectively). These inhibitory effects were abrogated when Fam65b mutants bear the additional RL mutation which precludes RhoA binding. Therefore, the inhibitory effect of Fam65b on T cell migration requires its ability to bind RhoA. Moreover, the non-phosphorylated and membranous form of Fam65b is the most efficient one to reduce T cell migration.

## Discussion

In this work, we show that Fam65b exerts a constitutive tonic inhibition on RhoA activation in resting T lymphocytes. Indeed, unstimulated T cells from Fam65b KO mice exhibit exacerbated levels of active RhoA. The purpose of this study was then to understand how the brake that Fam65b exerts on RhoA can be relieved upon chemokine stimulation to allow RhoA activation.

We show here that the key event is the phosphorylation of Fam65b by an as yet unidentified Ser/Thr kinase. The mode of Fam65b inactivation by stimulus-induced phosphorylation that we show here, is reminiscent of the one that operates on classical GDI proteins. Indeed, it has been shown that GDIa phosphorylation releases Rho GTPases that can then be activated by GEF at the plasma membrane (24, 25).

In T cells, Fam65b phosphorylation will have at least two connected consequences: (i) it directly decreases the affinity of its interaction with RhoA and consequently, (ii) it favors the shuttling of a fraction of Fam65b proteins from the plasma membrane to the cytosol.

The reason why non-phosphorylated Fam65b is localized at the plasma membrane is not clear yet. In addition to the requirement for RhoA binding that we show here, there are several hypotheses that could explain this particular distribution. It has been suggested that the Fam65b protein contains BAR domains, i.e., domains that promote membrane anchoring. This would explain why, in the absence of this domain, Fam65b has an essentially cytoplasmic localization (26). These domains are recognized by sequence homology with proteins that are known to have some. This method makes it possible to have an insight into the probability of existence of these domains. The presence of these BAR domains in Fam65b is nonetheless controversial (27, 28). To confirm the existence of such domains, it would be necessary to perform a crystallographic analysis of Fam65b, which would confirm or invalidate this hypothesis. Another hypothesis is that Fam65b binds to an unknown partner at the level of the plasma membrane and that phosphorylation of Fam65b alters its structure by the addition of a group creating a negative charge which would make the interaction between the two proteins weaker and release Fam65b from its membranous location.

Our results show that the WT form of Fam65b, which is known to be hypophosphorylated, is mainly located in the cytosol and inhibits RhoA activation. We also show that the mutant Fam65b (S9A) is not phosphorylated by chemokine stimulation and is localized mainly at the plasma membrane. Moreover, Fam65b (S9A) is more potent than Fam65b (WT) for binding RhoA and inhibiting the RhoA pathway, and thus the appearance of stress fibers in the HBMEC endothelial lineage and migration in the T CEM line. Since RhoA is generally considered to be activated at the level of the plasma membrane by GEFs, it would seem logical that a more widely membrane-bound form of Fam65b has a stronger inhibitory effect on the activation of the RhoA pathway which originates at the plasma membrane. Moreover, Fam65b (S9A) has a greater capacity than Fam65b (WT) to bind RhoA *in vitro*. This greater affinity could explain the fact that Fam65b (S9A) is also more effective than Fam65b (WT) in inhibiting the migration of T lymphocytes. Nevertheless, the inhibitory effect of these two Fam65b proteins on the formation of stress fibers and migration also depends on their ability to interact with RhoA since the introduction of the RL mutation in the Fam65b (RL) and Fam65b (S9A, RL) suppresses the inhibitory effect of Fam65b on the formation of stress fibers and on migration. This observation is consistent with the results previously published by our team showing that the Fam65b-RhoA interaction is direct.

We and others have shown that Fam65b interacts with the 14-3-3 chaperone protein (12, 21). 14-3-3 binds to the phosphorylated form of Fam65b, so it is likely that 14-3-3 will play a role in the regulation of the association of Fam65b and RhoA. Indeed, there may be competition between RhoA and 14-3-3 to bind Fam65b: non-phosphorylated form of Fam65b would preferentially bind to RhoA, whereas the phosphorylated form of Fam65b would tend to interact with 14-3-3, thus relieving the brake that Fam65b usually exerts on RhoA in resting cells. Fam65b could therefore be a new protein that takes part in the cross-talk between 14-3-3 and RhoA signaling pathways, as has been recently described for the interactions between the signaling pathways regulated by 14-3-3 and Rac1 (29).

Many actors of the cytoskeleton intervene, by more or less varied mechanisms, in the motility of cells and particularly of the T lymphocytes. The localization of Fam65b at the membrane could allow it to inhibit a membranous pool of RhoA and to allow fast activation of this pathway during a chemokinetic stimulation, since RhoA is already membranous and thus close to the exchange factors which activate it.

It is also necessary for a cell to tightly control the expression and activity of negative RhoA regulators such as Fam65b. Indeed, our results show that overexpression of Fam65b inhibits the RhoA signaling pathway, whereas knocking-out Fam65b greatly increases the levels of active RhoA. In both cases however, T cell migration is impaired, showing that Fam65b expression level is key to allow optimal T lymphocyte motility both *in vitro* and *in vivo*. Non-physiological levels of RhoA activity, either higher or lower, may cause similar cellular defects. This is a typical feature of Rho GTPases (30).

All these results are therefore in agreement with the model that, under basal conditions, Fam65b is associated with RhoA at the plasma membrane and that, after chemokine stimulation, Fam65b is phosphorylated, dissociates from RhoA and accumulates in the cytosol. The RhoA at the plasma membrane can then be activated by GEF and induce downstream signaling events required for T lymphocyte migration.

## Materials and methods

### Reagents

The calyculin A and Y27632 pharmacological inhibitors were purchased from Merck. CCL19 and CXCL12 (both from Peprotech) were used at the indicated concentrations.

### Constructs

Fam65b-iso2 was amplified by PCR from the pFam65b-V5 vector (31). The resulting PCR fragment was then introduced into the pEGFP-N1 vector (Clontech). Point mutants of Fam65b (RL: Arg151 and Leu152 in Ala and S9A: Ser 5, 21, 37, 166, 175, 341, 473, 474, 523 in Ala) were obtained by site-directed mutagenesis (QuikChange kit, Agilent Technologies). Plasmid pCAAX-DsRed encoding a red fluorescent protein capable of labeling the plasma membrane of living cells was provided by F. Niedergang (Cochin Institute).

### Cells

Peripheral Blood CD3^+^ T lymphocytes (PBTs) were purified from the blood of healthy donors provided by the *Etablissement Français du Sang* on Ficoll gradients before performing a negative selection sorting with the EasySep Human T cell Isolation kit (Stem Cell). The cells were then cultivated in RPMI medium supplemented with 10% of human AB serum, antibiotics (penicillin 50 U/ml, Streptomycin 50 μg/ml, Gibco), glutamine (4 mM, Gibco) and sodium pyruvate (1 mM, Gibco).

Murine CD3^+^ T lymphocytes were purified with the EasySep Mouse T cell Isolation kit (Stem Cell) using cell suspensions coming from the spleen and lymph nodes of WT or Fam65b KO mice. Before sorting, red blood cells present with splenocytes were lysed in the ACK buffer (155 mM NH_4_Cl, 10 mM KHCO_3_, and 1 mM EDTA, pH 7.3).

CEM cells (leukemic cell line) were cultured in RPMI medium supplemented with 10% fetal calf serum (Dominique Dutscher, France), sodium pyruvate (1 mM, Gibco), glutamine (4 mM, Gibco), and antibiotics (penicillin 50 U/ml, Streptomycin 50 μg/ml, Gibco). 293T cells and HBMEC (human bone marrow endothelial cells; gift from S. Bourdoulous, Cochin Institute) were cutured in DMEM containing the same additives.

### Fam65b knock-out (KO) mice

In order to obtain a model of mouse deficient for Fam65b, two lox sequences were first introduced by PCR on both sides of exons 3 and 4 of the *Fam65b* gene (Supplementary Figure 1A). Linearized targeting vector was transfected into ES cells. Homologous recombinants were identified by Southern-blot analysis, and were implanted into foster mothers. Chimeric mice were bred to C57BL/6J mice, and the F1 generation was screened for germline transmission. Mice carrying the floxed allele of *Fam65b* were backcrossed to C57BL/6J for five generations using the speed backcross technology (Charles River) to achieve 99.61% identity with C57BL/6J genome. Animals carrying such lox sites on their two alleles (Fam65b^lox/lox^ mice) were then crossed with transgenic mice expressing the Cre recombinase under the control of the CD4 promoter (provided by S. Amigorena, Institut Curie, Paris). The resulting Fam65b^lox/lox^ x Cd4-Cre animals give rise to mice exhibiting a T cell-specific deletion of Fam65b (Fam65b KO). Fam65b^lox/lox^ animals that do not express Cd4-Cre were used as controls (WT mice) in all the reported assays. For each experiment, the WT and Fam65b KO mice were systematically sex-matched and littermates. All mice were maintained under specific pathogen-free conditions. All animal experimentation was conducted in accordance with institutional guidelines.

### DNA transfections

3.10^6^ 293T cells per condition were transfected with 10 μg of plasmid DNA into 10 cm Petri dishes using lipofectamine 2000 (Invitrogen) according to the protocol provided by the supplier. The cells were used 48 h later for further experiments.

5.10^5^ HBMEC per cuvette were washed in PBS and resuspended in 100 μl of Cell Line Nucleofector Solutions V (Lonza). These cells were transfected by nucleofection using the U-015 program (Lonza) with 3–4 μg of DNA.

2.10^6^ or 5.10^6^ CEM cells per condition were washed in PBS and resuspended in 100 μl of Cell Line Nucleofector Solutions V (Lonza). These cells were transfected by nucleofection using the C-016 program (Lonza) with 5 or 10 μg of DNA, respectively.

10.10^6^ human primary T cells per condition were washed in PBS and resuspended in 100 μl of Human T Cell Nucleofector Solution (Lonza). These cells were transfected by nucleofection using the U-014 program (Lonza) with 10 μg of DNA.

All cells transfected by nucleofection were used for functional assays after an overnight incubation time.

### Western blots

Human PBTs or mouse T cells stimulated or not with 200 ng/ml CCL19 or CXCL12 (Peprotech) for 2 min were lysed for 30 min at 4°C in RIPA lysis buffer (50 mM Tris HCl pH 8, 150 mM NaCl, 0.1% SDS, 1% NP-40, 0.5% deoxycholate, 2mM EDTA) supplemented with a cocktail of protease inhibitors (EDTA-free Complete Inhibitors, Roche) and phosphatases (PhosSTOP, Roche). The nuclei and cell debris were removed by centrifugation at 14,000 rpm for 10 min at 4°C. The lysates were diluted in Laemmli buffer (500 mM Tris pH 6.8, 10% SDS, 10% glycerol, 5% β-mercaptoethanol, 10% bromophenol blue) and the proteins were then denatured by heating for 5 min at 100°C. Next, the samples were run on an 8 or 15% acrylamide gel. Molecular weight markers (Fermentas) were also run in parallel. The proteins separated by electrophoresis were next transferred on a PVDF membrane (GE Healthcare Life Sciences). After 1 h of saturation in T-TBS (0.1% Tween 20 (Sigma), 10% TBS 10X) containing 5% milk, the membrane was incubated overnight at 4°C with the following primary antibodies diluted in T-TBS containing 1% BSA: anti-β-actin (clone AC-74, Sigma), anti-GFP (rabbit polyclonal antibody # TP401, Torrey Pines Biolabs), anti-Fam65b (clone 2F6-1A11, Abnova), anti- Myosin Light Chain 2 (rabbit polyclonal antibody # FL172, Santa Cruz) and anti-p-Myosin Light Chain 2 (S19) (rabbit polyclonal antibody # 3671, Cell Signaling Technology). After 3 rapid washes in T-TBS with the SNAP system id. 2.0 (Millipore), the membrane was incubated for 10 min with the relevant goat anti-mouse or anti-rabbit secondary antibody coupled to HRP (# 115-035-146 or # 111-035-144, Jackson Laboratory). After aspiration of the solution containing the secondary antibody, the membrane was washed 3 times with T-TBS. The revelation was performed with an ECL kit (GE Healthcare Life Sciences). The emitted signal was detected by a camera (Fusion FX7, Vilbert Lourmat).

### Pull-down assays

GST pull-down experiments were performed on 293T cells or PBTs (50.10^6^ cells per condition) stimulated in 1 ml of HBSS containing 200 ng/ml CCL19 for 2 min or 200 nM Calyculin A for 10 min at 37°C. The cells were then lysed for 30 min at 4°C in pull-down buffer (100 mM NaCl, 50 mM Tris HCl, 1% NP-40, 10% Glycerol, 2 mM MgCl_2_) supplemented with protease inhibitors and phosphatases inhibitors in most experiments. After a 10 min centrifugation step at 14.000 rpm at 4°C, the supernatant was treated or not with Phosphatase λ (Merck) according to the manufacturer protocol. A fraction of the supernatant was then set aside as a control of whole cell lysate proteins and the rest of the supernatant was incubated with Sepharose beads coated with Glutathione (GE Healthcare) with shaking for 1 h at 4°C to eliminate protein non-specific binding to beads (pre-clearing step). In parallel, Glutathione beads were incubated with recombinant GST or GST-RhoA (Cytoskeleton) proteins in PBS with stirring for 1 h at 4°C. Alternatively, beads directly coupled to GST or GST-RhoA (Cytoskeleton) were also used. After washing the beads, the pre-cleared lysates were then incubated with the Glutathione beads loaded with GST or GST-RhoA for 2 h at 4°C under continuous rotation. The beads were then washed 3 times in pull-down buffer and diluted in Laemmli buffer. The proteins were next separated by SDS-PAGE and revealed by Western blotting with the following antibodies: anti-Fam65b, anti-GST (# 06-332, Upstate Biotechnology Inc.), anti-pERM (rabbit polyclonal antibody # 3141, Cell Signaling Technology) or anti-GFP.

### Phosphoproteomics

For phosphoproteomic experiments, 400.10^6^ PBT or 293T cells transfected with the isoform 2 of Fam65b-V5 were used. PBTs were stimulated at 50.10^6^/ml for 2 min with 200 ng/ml CCL19 at 37°C, and the cells were lysed for 30 min at 4°C in pull-down buffer supplemented with proteases and phosphatases inhibitors. After 10 min of centrifugation at 14,000 rpm at 4°C, the supernatant was incubated with 10 μg/ml anti-Fam65b antibody (clone 3J7, Santa Cruz) for PBTs, or an anti-V5 antibody (# R960-25, Invitrogen) for 293T cells. After 5 h of incubation with constant stirring at 4°C, magnetic beads coupled to Protein G or Protein A (Dynabeads, Invitrogen) for PBTs or 293T cells respectively, were added to the samples and incubated for 1 h with agitation at 4°C. The beads were then washed 3 times in pull-down buffer, and resuspended in Laemmli buffer. The proteins were separated by electrophoresis migration on an 8% acrylamide gel, stained with Coomassie blue, and the bands of interest were cut, processed and analyzed by Benjamin Thomas (Central Proteomics Facility, Sir William Dunn School of Pathology, University of Oxford, United Kingdom). Data were analyzed using PD1.4, Mascot search, PhosphoRS and Percolator. Protein coverage was 97% and only phospho sites whose probability was close to 100% was considered to be a phosphorylated amino acids.

### Cell fractionation

30.10^6^ PBT were resuspended in 1 ml of HBSS and stimulated in the presence of 200 ng/ml CCL19 for the indicated times at 37°C. The cells were then centrifuged, and the pellet resuspended in 200 μl of cold hypotonic buffer (10 mM KCl, 10 mM Hepes, 5 mM MgCl_2_) supplemented with proteases and phosphatases inhibitors, and 3.8 μm diameter beads (SPHERO Polystyrene Particles). The samples were then allowed to go through a syringe mounted with a 30 G needle eight times. After 10 min of centrifugation at 200 g and 4°C, the supernatant was transferred to a new tube and centrifuged for 45 min at 20000 g at 4°C. The supernatant containing the cytosolic fraction was recovered in a new tube and the protein concentration was measured. The pellet was resuspended in 50 μl of lysis buffer (20 mM Tris-HCl pH 7.5, 150 mM NaCl, 1% NP-40) supplemented with proteases and phosphatases inhibitors. After 20 min of incubation at 4°C with vigorous stirring and centrifugation for 45 min at 20,000 g at 4°C, the supernatant containing the membrane fraction was recovered, and the pellet resuspended in a solution of 1% SDS in H_2_O supplemented with proteases and phosphatases inhibitors. After 20 min incubation at 4°C with vigorous stirring, this solution was recovered. It constitutes the insoluble or cytoskeletal fraction. The proteins contained in these different fractions were then separated by SDS-PAGE and sequentially revealed by Western blotting with antibodies against Fam65b (clone 2F6-1A11, Abnova) and the following antibodies: Erk1/2 (rabbit polyclonal antibody # V1141, Promega) and CD3-ε (clone UCHT1, BD Biosciences) used as specific markers to control the purity of the cytosolic and membranous fractions, respectively. The amount of isoforms 1 and 2 of Fam65b in the different fractions was quantified by measuring the Fam65b bands intensities divided by the markers bands intensities in each condition, using the ImageJ software.

### Flow cytometry

For intracellular staining of active RhoA, T cells from WT or Fam65b KO mice were washed in cold PBS, fixed with 4% PFA (Electron Microscopy Sciences), centrifuged for 3 min at 3,000 rpm at 4°C and resuspended in permeabilization buffer (PBS, 0.5% saponin, 5% FCS, 4 mM NaN_3_) with 1/100 anti-RhoA-GTP antibody (# 26904, NewEast Biosciences). After 30 min of incubation at 4°C, the cells were washed in cold PBS containing 5% FCS and then incubated in permeabilization buffer with a secondary antibody directed against mouse IgG coupled to a fluorophore for 30 min at 4°C. After washing, the fluorescence of the cells was measured on a FACScan (Becton Dickinson). The staining for total RhoA was carried out in parallel with a similar protocol using an anti-RhoA antibody (# ARH03, Cytoskeleton).

### Microscopy

Transfected live CEM cells stained with DAPI were analyzed by fluorescence microscopy with a 60X immersion objective (Eclipse TE3200, Nikon). The images were collected using a cooled CCD camera (CoolSNAPfx, Ropper Scientific) and analyzed with the ImageJ software. Quantification of the subcellular localization of the Fam65b mutants was performed according to the formula: GFP fluorescence intensity in the cytoplasm (region that is neither stained with DAPI nor CAAX-DsRed)/GFP Intensity at the plasma membrane (region stained by the CAAX-DsRed Probe).

Transfected HBMEC cells were fixed with 4% PFA and then washed in washing buffer (PBS, 1% BSA). Next, the cells were permeabilized in PBS containing 0.1% saponin and 0.2% BSA, and then incubated for 45 min at room temperature with Phalloidine-TRITC (# P1951, Sigma) to stain the actin microfilaments. FluorSave (Calbiochem) was used as a mounting medium. For each transfectant, the number of stress fibers as well as the total F-actin staining intensity were quantified using ImageJ.

### 3D immunofluorescence

Twenty minutes after adoptive transfer, 3D immunofluorescence (3-DIF) was performed (15). Hundred microgram anti–L-selectin (clone Mel-14, Stem Cell) and 5 μg Alexa Fluor 633-conjugated anti-PNAd (clone MECA-79, BioLegend) were injected intravenously. Twenty minutes later, mice were killed using CO_2_, perfused with 5 ml cold PBS, and fixed in 10 ml cold 1% PFA/PBS. Peripheral lymph nodes (PLNs) were collected and manually cleaned from surrounding fat tissue under a stereomicroscope. After overnight fixation in PBS/1% PFA, PLNs were embedded into 2% (weight/vol) agarose low gelling point (Sigma), dehydrated in methanol for 24 h, and cleared for at least 2 days in benzyl alcohol-benzyl benzoate (1:2 ratio). Blocks of agarose-embedded PLNs were imaged using a 2PM setup (TrimScope; LaVision Biotec, Bielefeld, Germany). The absolute number of lymphocytes labeled with each dye was counted using Volocity software (PerkinElmer). Visual analysis of individual z-sections was used to determine the position of individual cells relative to the HEV network. After reconstruction of the lymph node in 3-DIF, the percentage of T cells inside or near the HEVs was also quantified as described (14). All animal work has been approved by the Cantonal Committee for Animal Experimentation and conducted according to federal guidelines.

### Transwell migration

CEM cells either pre-incubated with DMSO or 5 μM Y27632 (Calbiochem) overnight or transfected with different plasmids were deposited in the upper compartment of a Transwell insert (pore diameter: 5 μm; Nunc). The lower compartment contained no chemokine or 200 ng/ml CXCL12. After 3 h of incubation, the inserts were removed, an equal amount of calibration beads (Flow-Check Fluorospheres, Beckman Coulter) was deposited in the wells, and the number of cells having migrated to the lower compartment relative to the number of beads was quantified by flow cytometry (FACS Calibur, BD).

For Y27632 experiments, migration was quantified as the number of migrating cells/total cell number. For each transfectant, an eventual bias in cell migration due to Fam65b expression was quantified by calculating the following formula: migration index = % of migrating GFP^+^ cells/% of migrating GFP^−^ cells.

### Two-photon microscopy of popliteal lymph node

PLNs purified from WT or Fam65b KO mice were loaded with 3 μM CMFDA, 5 μM CMAC or 2.5 μM CMTMR fluorescent probes for 20 min at 37°C. 5.10^6^ cells of each population were then mixed with a 1:1 ratio and injected intravenously in a recipient wild-type C57BL/6J mouse. Twenty-four hours after injection, the popliteal lymph node was exposed and visualized by 2-photon microscopy (TrimScope system equipped with a 20X lens Olympus BX50WI fluorescence microscope). Images were acquired every 20 s for 20–30 min. The T cell zone was identified by the presence of HEV labeled after injection of 15 μg MECA-79-A633 antibody in the recipient mouse. The reconstruction and analysis of the images were performed with the software Volocity. Only T cell trajectories in the lymph node parenchyma were considered, and cells leaving or approaching the HEV were not taken into account in order to avoid abrupt changes of speed. Several parameters were quantified: migration speed, meandering index, directional changes and motility coefficient.

### Statistics

Significant differences between the groups were assessed using unpaired Student *t*-test or the Mann-Whitney test calculated with the Prism 5 software (GraphPad Software).

## Ethics statement

Healthy donors from Etablissement Français du Sang provided blood samples. Mice were handled according to the Paris Descartes University Ethics Committee and the European Union rules.

## Author contributions

LM, FM, JS, MM, and JD designed experiments. LM, EE, FM, MV, SC, NR, MM, and JD performed experiments. LM, EE, FM, MV, SC, NR, JS, MM, and JD performed data analysis. JD supervised the research project and wrote the manuscript with approval from all authors.

### Conflict of interest statement

The authors declare that the research was conducted in the absence of any commercial or financial relationships that could be construed as a potential conflict of interest.

## Supplementary material

Supplementary Figure 1 Generation of mice with T cell-specific deletion of the *Fam65b* gene. **(A)** Schematic presentation of original allele (+), targeting vector, the recombinant allele, the floxed allele (*Fam65b*) and the invalidated allele (*Fam65b*). The position of the forward (F) and reverse (R) primers used for genotyping are indicated as black arrows on the original allele. Filled blue boxes represent the exons 3 and 4 of the *Fam65b* gene. The locations of restriction-enzyme sites (XhoI, PacI, MluI, NotI and AscI) are indicated. Loxp and Frt sites are showed as red arrowheads and filled green boxes, respectively. The hygromycin resistance cassette is shown as a white box. **(B)** PCR genotyping of the floxed *Fam65b* allele and the original allele.

Supplementary Figure 2 Fam65b KO mice do not exhibit alterations in thymocyte and T cell numbers. **(A)** Western blot analysis of Fam65b expression in thymocytes and splenic T cells from WT or Fam65b KO mice. The anti-β-actin immunoblot was used as a loading control. **(B)** Similar Western blot analysis using splenic non-T cells from WT or Fam65b KO mice. **(C)** WT of Fam65b KO thymocytes and T lymphocytes purified from Peyer's patches, spleen, peripheral (p) or mesenteric (m) lymph nodes (LN) were counted. Each dot represents a single mouse.

Supplementary Figure 3 CXCL12 or CCL19 stimulation induces a shift of Fam65b bands. Western blot analysis of Fam65b isoforms 1 and 2 upon CCL19 or CXCL12 stimulation of human PBTs.

Supplementary Figure 4 Fam65b inhibits the RhoA signaling pathway. Top: HBMEC cells were transfected with expression vectors encoding GFP alone, Fam65b (WT), Fam65b(S9A), Fam65b(RL), or Fam65b(S9A, RL) all tagged with GFP. The cells were then labeled with phalloidin to visualize the actin filaments by microscopy. The representative images shown were acquired with a 60X magnification. Quantification of the number of stress fibers (bottom left) and F-actin staining intensity (bottom right) in HBMEC cells (20 ≤ n ≤ 30). ^**^*p* < 0.01, ^***^*p* < 0.001, and ^****^*p* < 0.0001.

Supplementary Figure 5 ROCK inhibition largely suppresses T cell migration. Quantification by flow cytometry of the percentage of CEM cells that have migrated through the Transwell insert in the presence or absence of Y27632 (ROCK inhibitor, gray bars) or DMSO (vehicle, black bars) upon stimulation (+) or not (–) with 200 ng/ml CXCL12. Means ± SE from three independent experiments. ^*^*p* < 0.05.
