# Supplementary figures and images for: Fam65b Phosphorylation Relieves Tonic RhoA Inhibition During T Cell Migration
Source: Front Immunol. 2018 Sep 11;9:2001. doi: 10.3389/fimmu.2018.02001 (PMC6141708; doi:10.3389/fimmu.2018.02001)

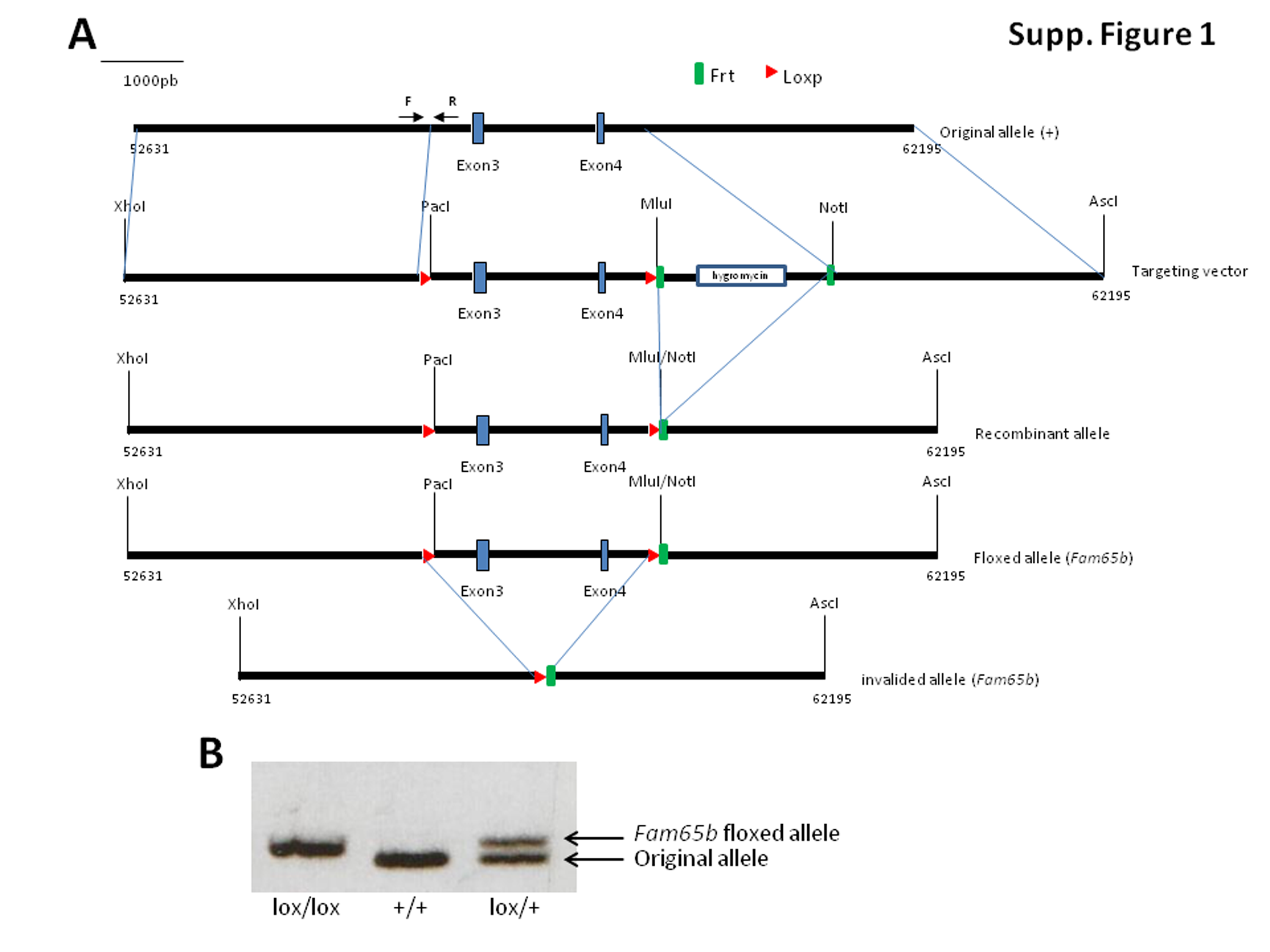

Supplement: Supplementary Figure 1 — Generation of mice with T cell-specific deletion of the Fam65b gene. (A) Schematic presentation of original allele (+), targeting vector, the recombinant allele, the floxed allele (Fam65b) and the invalidated allele (Fam65b). The position of the forward (F) and reverse (R) primers used for genotyping are indicated as black arrows on the original allele. Filled blue boxes represent the exons 3 and 4 of the Fam65b gene. The locations of restriction-enzyme sites (XhoI, PacI, MluI, NotI and AscI) are indicated. Loxp and Frt sites are showed as red arrowheads and filled green boxes, respectively. The hygromycin resistance cassette is shown as a white box. (B) PCR genotyping of the floxed Fam65b allele and the original allele. [file Image_1.tif]

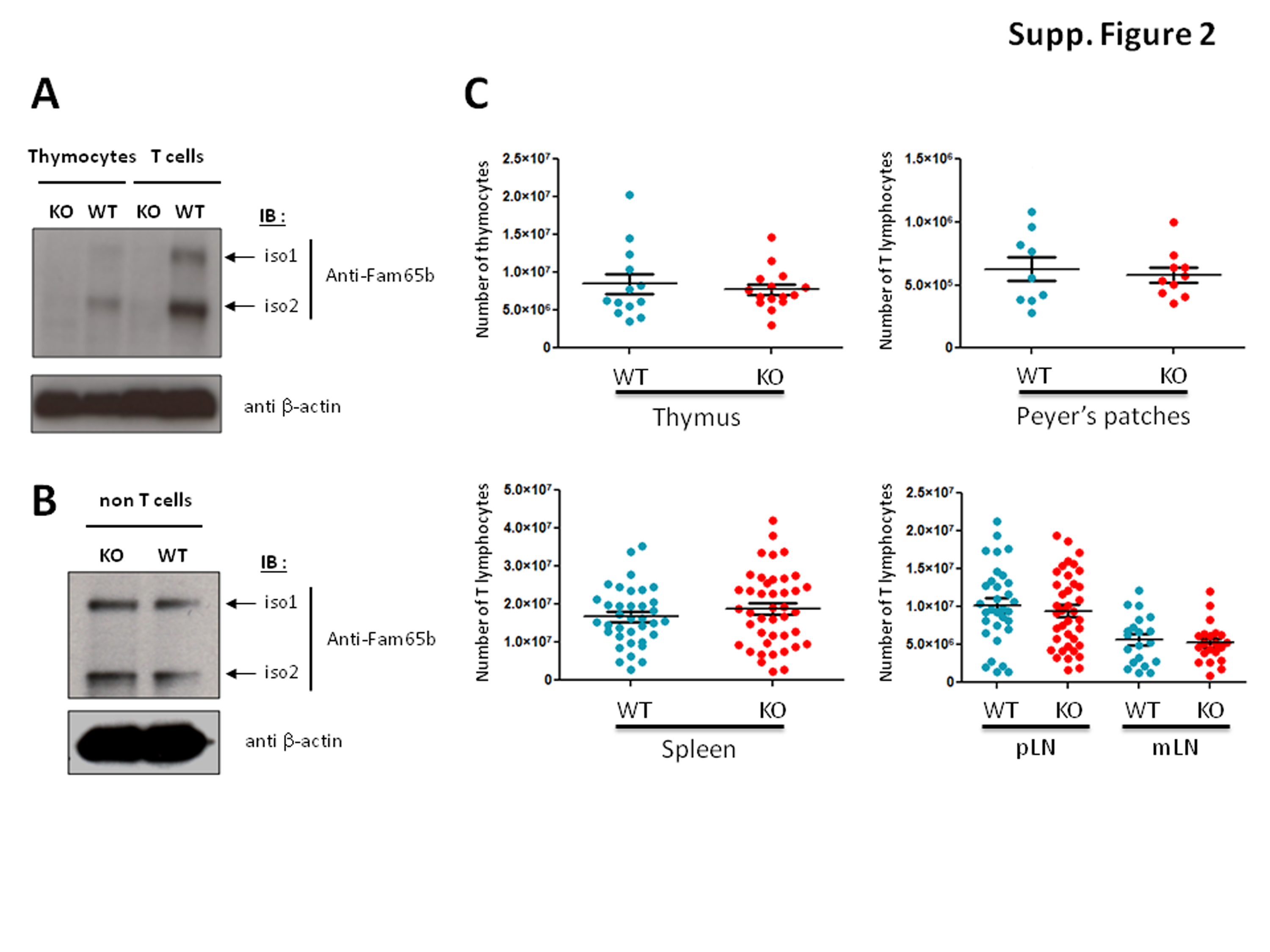

Supplement: Supplementary Figure 2 — Fam65b KO mice do not exhibit alterations in thymocyte and T cell numbers. (A) Western blot analysis of Fam65b expression in thymocytes and splenic T cells from WT or Fam65b KO mice. The anti-β-actin immunoblot was used as a loading control. (B) Similar Western blot analysis using splenic non-T cells from WT or Fam65b KO mice. (C) WT of Fam65b KO thymocytes and T lymphocytes purified from Peyer's patches, spleen, peripheral (p) or mesenteric (m) lymph nodes (LN) were counted. Each dot represents a single mouse. [file Image_2.tif]

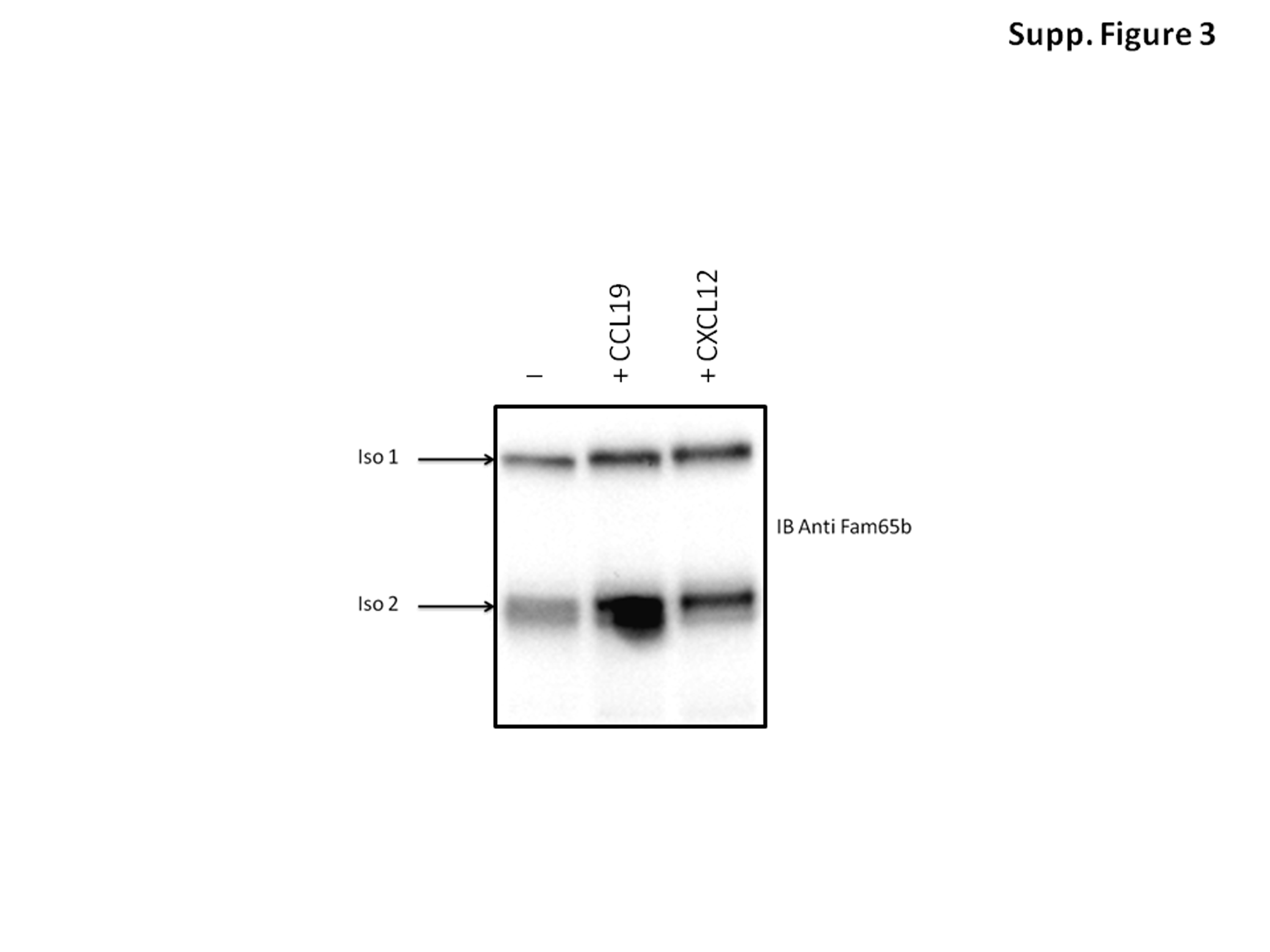

Supplement: Supplementary Figure 3 — CXCL12 or CCL19 stimulation induces a shift of Fam65b bands. Western blot analysis of Fam65b isoforms 1 and 2 upon CCL19 or CXCL12 stimulation of human PBTs. [file Image_3.tif]

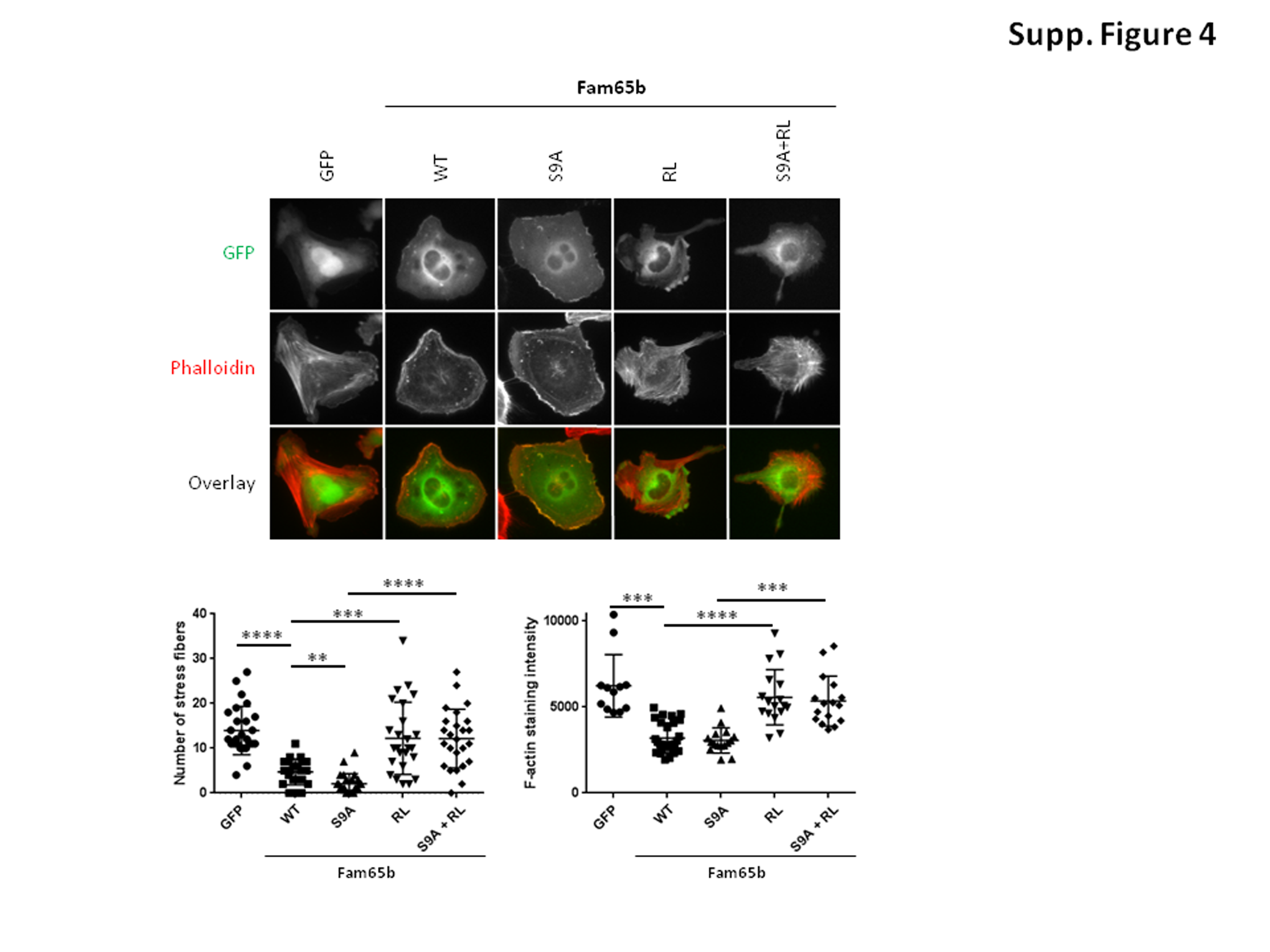

Supplement: Supplementary Figure 4 — Fam65b inhibits the RhoA signaling pathway. Top: HBMEC cells were transfected with expression vectors encoding GFP alone, Fam65b (WT), Fam65b(S9A), Fam65b(RL), or Fam65b(S9A, RL) all tagged with GFP. The cells were then labeled with phalloidin to visualize the actin filaments by microscopy. The representative images shown were acquired with a 60X magnification. Quantification of the number of stress fibers (bottom left) and F-actin staining intensity (bottom right) in HBMEC cells (20 ≤ n ≤ 30). **p < 0.01, ***p < 0.001, and ****p < 0.0001. [file Image_4.tif]

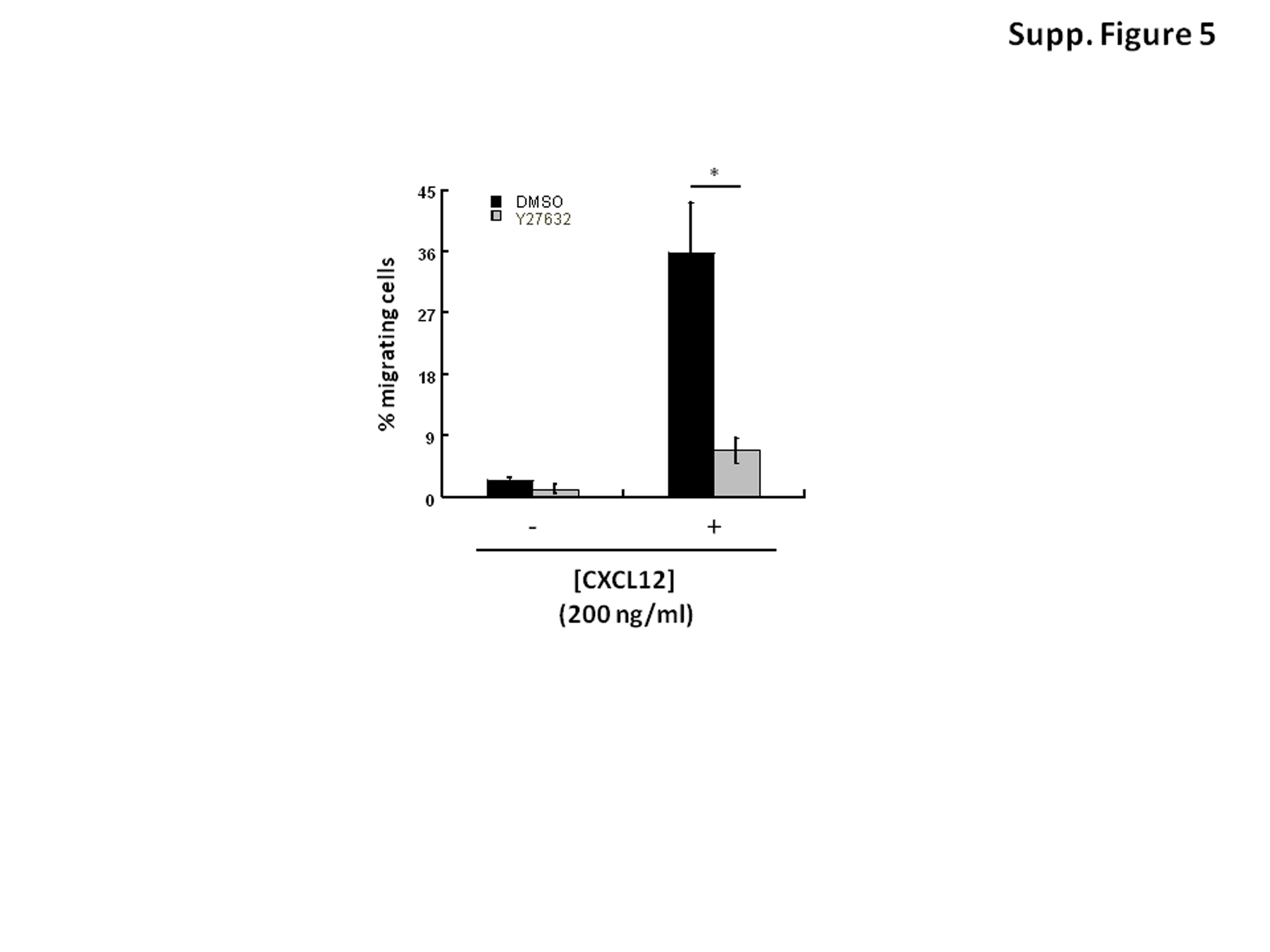

Supplement: Supplementary Figure 5 — ROCK inhibition largely suppresses T cell migration. Quantification by flow cytometry of the percentage of CEM cells that have migrated through the Transwell insert in the presence or absence of Y27632 (ROCK inhibitor, gray bars) or DMSO (vehicle, black bars) upon stimulation (+) or not (–) with 200 ng/ml CXCL12. Means ± SE from three independent experiments. *p < 0.05. [file Image_5.tif]
